# Validity and reliability of social anxiety disorder diagnoses in the Swedish National Patient Register

**DOI:** 10.1186/s12888-020-02644-7

**Published:** 2020-05-15

**Authors:** Alba Vilaplana-Pérez, Josef Isung, Sonja Krig, Sarah Vigerland, Maral Jolstedt, Johan Bjureberg, Jens Högström, Kayoko Isomura, Daniel Rautio, Eva Serlachius, Christian Rück, David Mataix-Cols, Lorena Fernández de la Cruz

**Affiliations:** 1 Centre for Psychiatry Research, Department of Clinical Neuroscience, Karolinska Institutet, Gävlegatan 22 (Entré B), floor 8, SE-11330 Stockholm, Sweden; 2 Stockholm Health Care Services, Region Stockholm, Stockholm, Sweden; 3 Departament de Personalitat, Avaluació i Tractaments Psicològics, Universitat de València, València, Spain

**Keywords:** Social anxiety disorder, Social phobia, Validity, Reliability, Epidemiology

## Abstract

**Background:**

Population-based administrative registers are often used for research purposes. However, their potential usefulness depends on the validity of the registered information. This study assessed the validity of the recorded codes for social anxiety disorder (SAD), also known as social phobia, in the Swedish National Patient Register (NPR).

**Methods:**

The personal identification numbers of 300 randomly selected individuals with a diagnosis of SAD recorded in the NPR were obtained from the Swedish National Board of Health and Welfare. The medical files of these individuals were then requested from clinics nationally. A total of 117 files were received and two independent raters reviewed each file to assess the presence or absence of SAD, according to the definition of the International Classification of Diseases, Tenth Edition (ICD-10) and the diagnostic criteria of the Diagnostic and Statistical Manual of Mental Disorders, Fourth Edition, Text Revision (DSM-IV-TR). When disagreements between the two raters were found, a third rater reviewed the file to establish a best estimate diagnosis. Positive predictive values (PPV) and agreement between the two initial raters (using Cohen’s kappa) were calculated. Additionally, raters completed the Clinical Global Impression – Severity (CGI-S) and the Global Assessment of Functioning (GAF) rating scales for each file. Inter-rater agreement for the CGI-S and the GAF was assessed using intraclass correlation coefficients (ICC).

**Results:**

After exclusion of files not containing sufficient information, 95 files were included in the analyses. Of these, 77 files (81.05%) were considered to be ‘true positive’ cases (PPV = 0.81, 95% confidence interval = 0.72–0.88). Inter-rater agreement regarding the presence or absence of SAD was substantial (κ = 0.72). CGI-S and GAF scores indicated that patients were in the moderate range of severity and functional impairment. Inter-rater agreement for the CGI-S and the GAF was moderate to good (ICC = 0.72 and ICC = 0.82, respectively).

**Conclusions:**

The ICD-10 codes for SAD in the Swedish NPR are generally valid and reliable, but we recommend sensitivity analyses in future register-based studies to minimise the impact of potential diagnostic misclassification. Most patients were moderately severe and impaired, suggesting that results from register-based studies of SAD may be generalizable.

## Background

Social anxiety disorder (SAD), also commonly known as social phobia, is one of the most common psychiatric disorders, with an average worldwide lifetime prevalence of 4% [24]. The disorder is associated with substantial functional impairment [1, 18] and presents with a remarkably high degree of comorbidity [2], mainly other anxiety disorders, mood disorders, substance use disorders, and impulse control disorder [21, 24]. While much is known about the clinical features and treatment of SAD, there are important gaps in our understanding of its aetiology and long-term medical and socioeconomic consequences [4].

Swedish nationwide registers – which contain administrative records from entire population ‘from cradle to grave’ – and a wealth of high-quality healthcare data prospectively collected over several decades, provide unique opportunities to study risk factors as well as the long-term consequences of psychiatric disorders. In 1964, the Swedish National Board of Health and Welfare started the National Patient Register (NPR). This register contains clinical diagnoses by medical specialists, together with administrative data such as hospital or clinic of treatment, dates of admission and discharge, surgical procedures, and patient characteristics including age, sex, and place of residence [16]. At first, the NPR only compiled somatic inpatient care data from six out of 26 Swedish counties, until 1969, when it was complemented with information from psychiatric inpatient units. Since 1984, a mandatory participation for all county councils allowed to connect all data through a 10-digit unique personal identity number given to every Swedish resident, enabling cross-linkage with a range of national registers [17]. Since 2001, the register also includes all outpatient visits from private and public medical doctors (including day surgery and psychiatric care, but excluding primary care). Diagnoses in the NPR are coded according to the Swedish International Classification of Diseases (ICD) system, which was adapted from the World Health Organization ICD classification system [16].

A large variety of epidemiological and genetic studies of psychiatric disorders have been conducted using the NPR, including SAD [11, 15, 23]. However, the usefulness of this research depends on the diagnostic validity of the registered cases [19]. A review showed that the accuracy of a range of diagnostic codes in the NPR, mainly somatic diseases, ranged from 85 to 95% [16]. In psychiatry, the validity of some diagnostic codes, such as obsessive-compulsive disorder [20], chronic tic disorders [20], schizophrenia [5], bipolar disorder [22], and autism spectrum disorders [10] has been established, while the validity of other diagnoses such as SAD, has not yet been studied.

The aim of this study was to facilitate further epidemiological research using the Swedish NPR by examining the diagnostic validity and reliability of recorded diagnoses for SAD. Additionally, because it cannot be assumed that the patients in the NPR are representative of the general population of individuals with SAD, we rated their symptom severity and global functioning.

## Methods

### Procedures

**Fig. 1:**
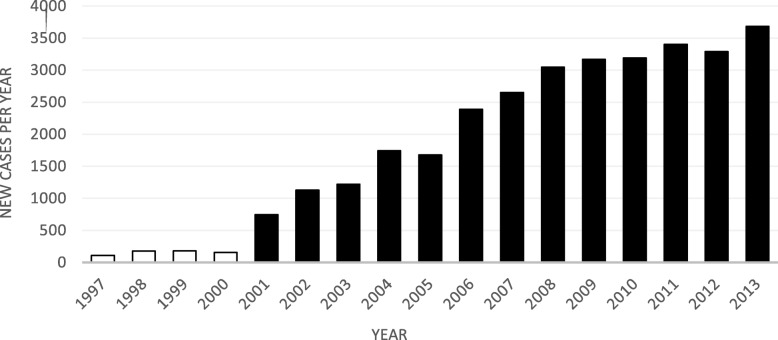
Annual incidence of social anxiety disorder in the Swedish National Patient Register during the period from 1997 (introduction of the ICD-10 codes in Sweden) and 2013. *Note:* White bars represent the number of new cases diagnosed from 1997 to 2000 (inpatients only); black bars represent the number of new cases diagnosed from 2001 to 2013 (inpatients and outpatients)

The study was approved by the regional ethical review board in Stockholm (2012/570–31/1). A request was sent to the Swedish National Board of Health and Welfare to obtain a random sample of 300 personal identification numbers with a record of a SAD diagnosis in the NPR who had been diagnosed anywhere in Sweden. The selection of random cases was undertaken by the Swedish National Board of Health and Welfare without any control or involvement from the study researchers. No weighting or any other adjustments were used to select the cases. The number of requested cases (*n* = 300) was decided on the basis of the response rates in a previous validation study and with the aim of reaching at least 100 cases for analyses [20]. From years 1997 to 2013, a total of 31,975 SAD cases were registered in the NPR, with more than 3000 new cases per year from 2008. The graphic representation of the annual incidence of SAD cases in the NPR is shown in Fig. 1. The steep increase from 2001 was due to the inclusion of the outpatient care services in the NPR.

**Fig. 2:**
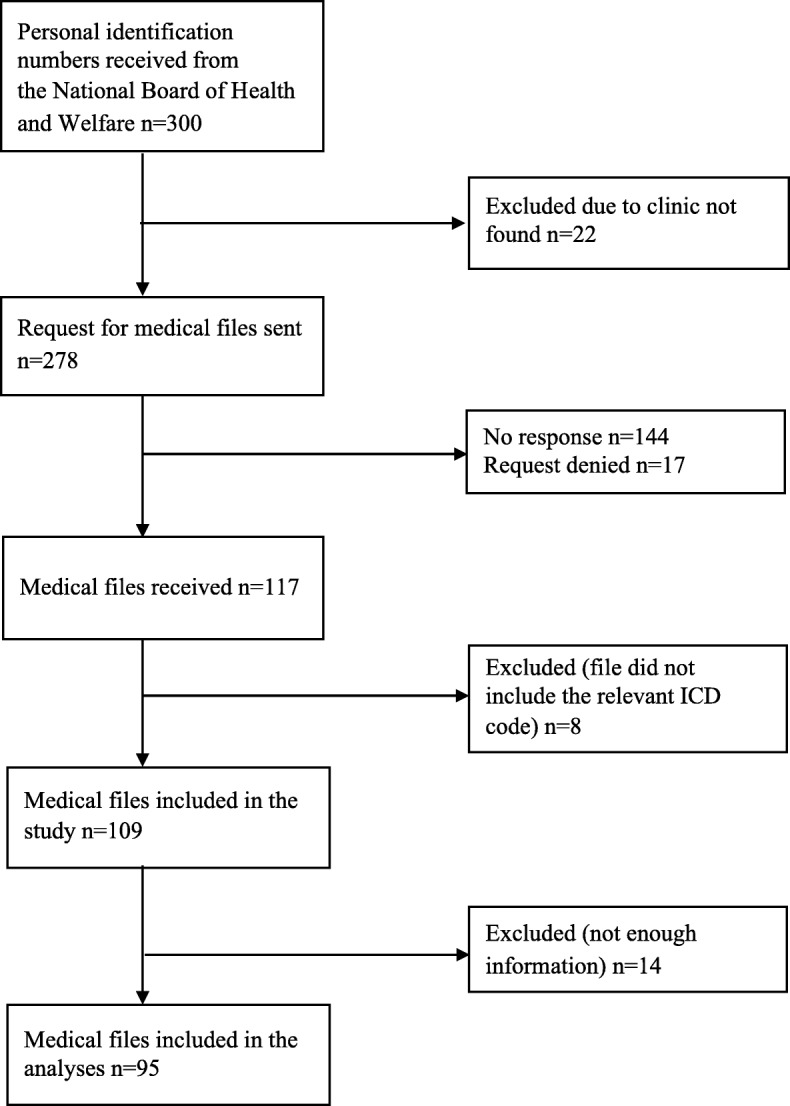
Flowchart of requested and received patient files containing a social anxiety disorder diagnosis code

In order to identify records of SAD, we used the 10th revision of the ICD (ICD-10) code (F40.1), since previous revisions of the manual did not include an independent code for SAD (or ‘social phobia’, as named in ICD-10). The dates of registered diagnosis spanned from 1998 to 2016 for those with diagnoses in the inpatient register, and from 2001 to 2016 for those in the outpatient register. An ICD-10 diagnosis of SAD at any time during this time period was sufficient to be eligible for inclusion. The vast majority of the medical files comprised comorbid disorders and therefore contained several ICD psychiatric diagnoses codes. Administrative data including the code of the hospital or clinic where the diagnosis was given was received, together with the personal identification numbers. The full medical files of the randomly selected cases were requested from the corresponding hospitals and clinics via written letters sent through regular mail. For 22 of the 300 cases, we were not able to locate an associated clinic (e.g., the available clinic code corresponded to a clinic that was no longer operative), and therefore requests were only sent for 278 cases. In 144 of these cases, the associated clinics did not reply to the written request, and in 17 additional cases, the associated clinics declined participation. Thus, files for 117 cases were received. Of these, eight cases were excluded after inspection of the patient record, as the SAD diagnosis code was not documented in the actual received file. In a final step, 14 further cases were excluded since there was not enough information in the available material to make a diagnostic judgement (e.g., the diagnostic code was written in the patient record but clinical notes describing symptomatology were not available). Therefore, the total number of available cases for review was 95. These procedures are similar to those used in previous validation studies using the NPR [10, 20]. Figure 2 shows the flowchart of cases included in the study.

### Chart review

Each of the 95 medical files available for analysis was assessed by two independent raters using a predefined score sheet (available from the corresponding author upon request). The group of raters performing the chart review was composed of 5 clinical psychologists and 2 psychiatrists, all but one with a PhD degree, with several years' experience in the assessment and treatment of anxiety disorders.

Raters were independently asked whether the information contained in the patient file was consistent with a probable diagnosis of ‘social phobia’ in the ICD-10. Further, since the ICD-10 classification does not contain operational diagnostic criteria but, instead, a narrative description of the disorder, raters were further asked whether the individual diagnostic criteria for ‘social phobia’ were also likely to be met according to the Diagnostic and Statistical Manual of Mental Disorders, Fourth Edition, Text Revision (DSM-IV-TR), allowing for a more systematic and operational evaluation. These judgements were largely based on the direct chart descriptions of significant levels of anxiety in social situations (e.g., nervousness in school or at work related to public speaking, anxiety when attending social events, trouble in socializing with others) and/or descriptions of avoidance of social situations due to these symptoms. Additionally, descriptions of social networks (or the lack of them), difficulties with dating or intimate relationships, evidence of academic or work underachievement or amount of sick leave linked to social anxiety symptoms were essential to assess the symptom severity and degree of functional impairment. When there were disagreements between the two raters regarding the definite or probable presence/absence of SAD, a third independent rater made a final judgement on the diagnostic status of the case. When raters considered that a case did not meet SAD criteria (i.e., false positive), they were asked to provide the most plausible alternative diagnosis.

Further, in order to assess SAD symptom severity and global functioning in our sample of cases, raters completed the Clinical Global Impression – Severity (CGI-S) [9] and the Global Assessment of Functioning (GAF) [7] rating scales. The CGI-S is a one-item measure (*“Considering your total clinical experience with this particular population, how mentally ill is the patient at this time?”*) which evaluates the severity of psychopathology (in this case, SAD symptoms) from 1 to 7, where 1 is ‘normal’ and 7 is ‘among the most extremely ill patients’. This measure has been previously validated for assessing severity of SAD cases [26]. The GAF is also a one-item scale (scores ranging from 1 through 100) used by mental health professionals to subjectively rate the general social, occupational, and psychological functioning of adults [12], where the range 91–100 indicates no symptoms that impair functioning (i.e., superior functioning) and the range 1–10 implies an extremely low functioning with persistent danger for self or others. The GAF has shown good validity and reliability in the assessment of overall functioning in psychiatric patients [12]. Both of these scales are generally rated in reference to the time of the evaluation (or the week before). In this case, and for the purposes of the study, raters were asked to rate these measures averaging the severity and functioning of the patient for the whole time frame covered in each of the assessed files.

### Statistical analyses

We calculated the positive predictive value (PPV) of the SAD diagnosis, that is, those cases diagnosed correctly divided by the sum of the true positives (e.g., the file was labelled as SAD and the raters agreed on the diagnosis) and the false positives (i.e., the file was labelled as SAD but the raters did not agree with this judgement), with their corresponding 95% confidence intervals (CIs). When rater 1 and rater 2 did not agree on the diagnosis, the judgement of a third rater was used as the best estimate diagnosis against the diagnosis in the file. Inter-rater reliability was calculated using the Cohen’s kappa statistic [3] with the ratings of the two initial raters. To assess the inter-rater agreement for the CGI-S and the GAF scales, intraclass correlation coefficients (ICC) with 95% CIs were calculated based on a mean-rating (k = 7), average measures, and 2-way mixed-effects model [13]. SPSS statistical package version 25 (SPSS Inc., Chicago, IL) was used for all the analyses.

## Results

### Validity and reliability of SAD codes in the NPR

A total of 95 cases (53 females, 56%), all from psychiatric clinics across the country, were included in the analyses. Of these, 77 (81%) were deemed as ‘true positive’ cases since raters considered that either the ICD-10 definition or the DSM-IV-TR diagnostic criteria for SAD were met. In most cases (94%), criteria were met according to both diagnostic systems. In the small number of cases where there was a discordance between diagnostic systems, raters considered that the ICD-10 definition was met, but not the most stringent DSM-IV-TR criteria.

The 77 ‘true positive’ cases corresponded to a PPV of 0.81 (95% CI, 0.72–0.88). The remaining 18 cases were not considered to fulfil neither ICD-10 nor DSM-IV-TR criteria for SAD and were, therefore, considered false positive cases. The most frequent alternative diagnoses were other anxiety disorders, depression, and autism spectrum disorders (Table 1).

**Table 1:** Alternative diagnoses for false positive cases (*n* = 18)

Alternative diagnoses	Frequency
Anxiety or post-traumatic stress disorder (other than social anxiety disorder)	7
Depression	5
Autism spectrum disorder	3
Bipolar disorder	2
Eating disorder	2
Attention-deficit/hyperactivity disorder	1
Psychotic disorder	1
Borderline personality disorder	1

*Note:* Numbers do not add up to the total of false positive cases (n = 18) since, for some cases, raters suggested more than one alternative diagnosis

The Cohen's kappa between the two initial raters regarding the presence or absence of a social anxiety disorder was 0.72. Of the 7 cases where there was a disagreement between raters, four cases were added to the final number of true positives (i.e., the third rater considered that the SAD diagnosis was present) and three were deemed false positives (i.e., the third rater considered that the SAD diagnosis was absent) after the best estimate diagnosis review.

**Fig. 3:**
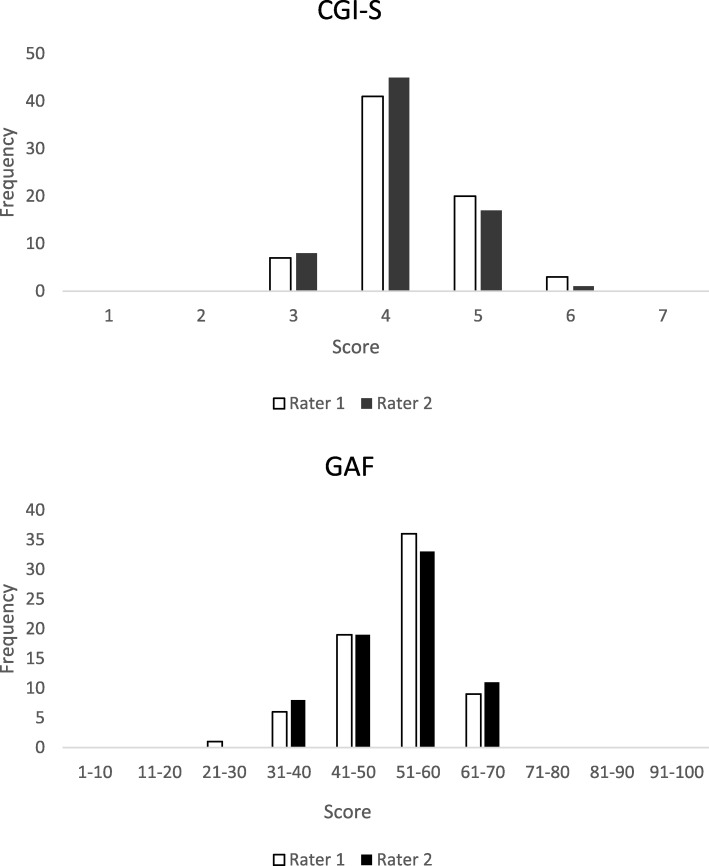
Score distribution of the Clinical Global Impression – Severity (CGI-S) and Global Assessment of Functioning (GAF), by rater

Of the 77 ‘true positive’ cases, 71 had obtained a CGI-S and GAF score from the raters (in the remaining six cases, raters had not scored the scales and therefore this information was missing). Regarding the CGI-S, the mean score was 4.27 (sd = 0.70) for rater 1 and 4.15 (sd = 0.62) for rater 2, indicating moderate severity of the assessed cases (Fig. 3). The inter-rater agreement for the CGI-S was moderate (ICC = 0.72 [95% CI, 0.54–0.82]). For the GAF, the mean score was 52.1 (sd = 8.77) for rater 1 and 53 (sd = 9.0) for rater 2, indicating moderate difficulty in social, occupational, or school functioning (Fig. 3). The inter-rater agreement for the GAF was good (ICC = 0.82 [95% CI, 0.71–0.89]).

## Discussion

The Swedish NPR includes more than 30,000 individuals with a diagnosis of SAD until 2013, and approximately 3000 new patients are added to the register each year. Therefore, the NPR constitutes a potentially unique resource for high quality epidemiological research into this disorder. However, before such research can be conducted, it is important to formally validate the corresponding ICD codes, which mainly rely on clinicians’ judgements and therefore may be biased or inaccurate. Reassuringly, our results showed that the diagnostic validity of SAD in the NPR is generally good, with a PPV of 0.81 when rating the overall occurrence of SAD through chart review, the gold standard method for confirming diagnoses [25]. Further, the inter-rater agreement for the disorder was substantial [14]. The validity of the SAD diagnosis is comparable to that of other psychiatric disorders in the NPR, including bipolar disorder (PPV = 0.81–0.91) [22], schizophrenia (PPV = 0.91–1.0) [6], obsessive-compulsive disorder (PPV = 0.55–0.96) [20] or tic disorders (PPV = 0.86–0.97) [20].

In our sample, 19% of the cases were regarded as false positives (i.e., expert raters did not agree with the recorded diagnoses). The most frequent alternative diagnoses were other anxiety disorders and mood disorders, which share clinical features with SAD, as well as the underlying dimensions of distress or negative affect, shared genetic predisposition and neurobiology [8]. Since misclassification was not uncommon, we recommend that future register-based studies of SAD be always accompanied with sensitivity analyses whereby different comorbidities are systematically excluded to evaluate their impact on the outcomes of interest.

Since the NPR only includes diagnoses given by specialists, it is often assumed that patients included in this register are at the most severe end of the spectrum, potentially affecting the generalizability of the register-based results to other populations (e.g., those being seen in primary care settings). However, our severity and global functioning measures were fairly normally distributed, with most patients belonging to the moderately ill category of the CGI-S and the moderate difficulty in social, occupational or school functioning of the GAF. Thus, the results of register-based studies of SAD are likely to generalise reasonably well to other treatment-seeking populations.

The main strength of this study is the random selection of cases diagnosed with SAD from clinics placed all over the country. The medical files were meticulously evaluated by independent and skilled clinical psychologists or psychiatrists in accordance with both ICD-10 descriptions and DSM-IV-TR diagnostic criteria for SAD. However, the study is not without limitations. First, we were only able to collect and therefore assess about one third of the initially requested cases, which may suggest a potential selection bias of unknown nature. Potential differences (e.g., in demographic or clinical variables) between the available files and those that could not be obtained could not be examined since individual information (beyond the personal identification number, the year of diagnosis, and the clinic that assigned the diagnosis) was not made available to us because it was not covered by our ethical approval for this study. However, the main reasons why we did not receive the files were mainly practical (e.g., some of the clinics did no longer exist, had confidentiality concerns or no administrative personnel was available to copy and post the files). Thus, although we cannot be certain, we believe that systematic bias is unlikely. Additionally, some of the files received did not contain sufficient information to make a decision on diagnosis; based on previous studies using similar methods [10, 20], we decided to exclude these files from the analyses, as considering them as either true positives or false positives would carry a substantial risk of reporting inaccurate PPVs. Second, because the study did not include control groups (i.e., medical records from patients without SAD), the raters were not blind to the register diagnoses, which may have increased the risk of bias towards confirming the SAD diagnoses. Lastly, raters scored the CGI and the GAF based on the chart review, without directly interviewing the patients. The validity of these scales when used in this format is unknown and, therefore, the results should only be viewed as broad clinical impressions of the patients’ severity and general function. Nonetheless, the inter-rater agreement was adequate for both instruments.

## Conclusions

The ICD-10 codes for SAD cases in the Swedish NPR are generally valid and reliable but we recommend sensitivity analyses in future register-based studies to minimise the impact of potential diagnostic misclassification. Most patients were moderately severe and impaired, suggesting that the results of register-based studies of SAD may be generalizable to other treatment-seeking populations.

## Data Availability

No additional data are available.

## Abbreviations

CGI-S: Clinical global impression – severity
DSM-IV-TR: Diagnostic and Statistical Manual of Mental Disorders, Fourth Edition, Text Revision
GAF: Global assessment of functioning
ICC: Intraclass correlation coefficients
ICD-10: International Classification of Diseases, Tenth Edition
NPR: National patient register
PPV: Positive predictive value
SAD: Social anxiety disorder
